# Isothermal Equation of State of Polyether Ether Ketone (PEEK) by Optical Imaging Method in Diamond Anvil Cell

**DOI:** 10.3390/polym17050655

**Published:** 2025-02-28

**Authors:** Ying Chen, Ning Yang, Yi Zhang, Lei Liu

**Affiliations:** 1 National Key Laboratory of Shock Wave and Detonation Physics, Institute of Fluid Physics, CAEP, Mianyang 621900, China; chenying22@gscaep.ac.cn (Y.C.); zhangyishp@126.com (Y.Z.); 2 Institute of Machinery Manufacturing Technology, CAEP, Chengdu 621200, China; yangning0706@126.com

**Keywords:** PEEK, high pressure, equation of state, diamond anvil cell

## Abstract

Polymers serve as important functional materials in various environments, including high-pressure conditions. However, the behavior of polymers under high pressure is currently less understood. In this study, the isothermal equation of state of polyether ether ketone (PEEK), an important polymer, was measured using the diamond anvil cell technique at up to 8 GPa. The isotropic compression behavior of PEEK samples was investigated by monitoring the area change in PEEK disks during the compression process using the optical imaging method. The present results shed light on the mechanical properties of PEEK under extreme conditions, which will guide the applications of PEEK at high pressures.

## 1. Introduction

Polymeric materials are one of the most important types of materials in modern society, with a wide range of applications in agriculture, the automotive industry, biomedicine, electronics, aerospace, energy, defense, and many other fields [1,2]. There is no doubt that polymers are becoming increasingly widely used and have become the answer to many of the challenges faced in everyday life [3]. In fields such as the automotive, aerospace, and defense industries, polymers face ever-complicated application environments, such as high-temperature and high-pressure conditions [4]. Polyether ether ketone (PEEK) is a high-performance engineering polymer material with excellent mechanical properties [5], heat resistance [6], and chemical stability [7], making it highly promising for use in cutting-edge industries [8]. With a maximum crystallinity of approximately 40%, PEEK exhibits a glass transition temperature (*T*_g_) of 143 °C, an infinite polymer melting temperature (*T*_m∞_) of around 390 °C, and a pyrolysis temperature of 550 °C [9]. These characteristics position PEEK as an ideal candidate for applications where high-temperature resistance and mechanical strength are paramount.

The equation of state (EOS) is an important thermodynamic property of materials, which describes the relationship between the fundamental thermodynamic variables of a system, such as volume (*V*), pressure (*P*), and temperature (*T*) [10,11]. At a constant temperature, the pressure–volume relations of a solid can be described by different types of analytical EOSs [12], which incorporate the isothermal bulk modulus [${K_{0}=-V\cdot \left(\frac{\partial P}{\partial V}\right)}_{T}$] and its pressure derivatives ($K_{0}^{\prime }=\frac{\partial K}{\partial P}$). The bulk modulus (*K*_0_) describes the resistance of the material to compression at ambient pressure, while the pressure derivative of the bulk modulus ($K_{0}^{\prime }$) characterizes the sensitivity of the bulk modulus to changes in pressure. For PEEK, the EOS can provide insights into its behavior under varying pressure conditions, which is essential for applications involving high-pressure environments, such as in the aerospace and the automotive industries.

There are several different techniques for measuring the EOS of materials, including X-ray diffraction (XRD), Brillouin scattering, X-ray tomography [13], and so on. XRD is the predominant technique employed in diamond anvil cells (DACs) to determine the EOS for crystalline materials, while it is invalid for amorphous materials [14]. Brillouin scattering is a typical inelastic scattering process, which is characterized by its very low signal intensity, especially at high pressures [15]. X-ray tomography requires a high-quality X-ray source, as well as a high density contrast between the sample and the surrounding materials (e.g., pressure-transmitting medium in DAC). PEEK is a polymer that exhibits both crystalline and amorphous phases with relatively low density (~1.26–1.30 g/cm^3^), presenting a complex molecular structure. Consequently, it is quite challenging to measure the EOS of PEEK at high pressures using the techniques mentioned above. 

Scott and Jeanloz first proposed the visible-light imaging technique to determine the EOS of a sample in DAC. They verified the technique by measuring the EOS of Au [16]. The visible-light imaging method is particularly suitable for measuring the EOS of opaque or amorphous materials such as PEEK due to its advantages of being non-destructive, having a high resolution, and being able to carry out automated analysis. This method is able to accurately capture the size change of the sample under high-pressure conditions, thereby directly deriving the volume strain, without the need for complex equipment, and it is compatible with DACs technology. Therefore, the EOS for PEEK can be derived from optical measurements conducted in a diamond anvil cell (DAC), which has become an effective tool for studying the behavior of materials under extreme conditions. 

The development and refinement of the DAC technique have significantly expanded the accessible thermodynamic space in high-pressure research. This technique has become a standard and powerful experimental method, routinely employed in in-house laboratories and at synchrotron beamlines [17]. Recent advancements in the DAC technique have enabled the generation of pressures reaching hundreds of GPa, or even a TPa magnitude [18,19], facilitating the study of materials under conditions that were previously unattainable. These high-pressure experiments offer a unique opportunity to explore the isothermal EOS of PEEK, particularly its bulk modulus, and thermal expansion coefficients.

In this work, the isothermal EOS of PEEK was investigated by using an optical imaging technique in DACs. By examining pressure–volume relationships, we contribute to a more comprehensive understanding of PEEK’s behavior under high-pressure conditions. This research is not only relevant to the fundamental science of polymer physics but also has practical implications for the engineering and design of PEEK components in high-performance applications. The insights gained are expected to inform material design and optimize performance in extreme conditions, potentially expanding PEEK’s utility in cutting-edge industries.

## 2. Experiment and Methods

### 2.1. Instrumental Setup Overview

**Figure 1:**
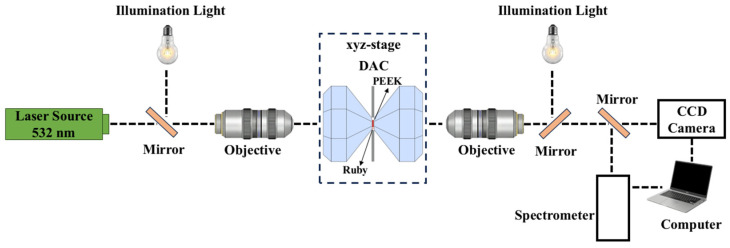
Schematic of the optical system used for measuring the equation of state of polymers.

In our experiments, the DAC optical imaging experimental setup consists of an LED transmission illumination device, a motorized XYZ sample stage, a microscopic amplification system, and a sample system, as shown in Figure 1. The image of the sample is formed by transmitted light through the DAC sample cavity and collected from the opposite side by a long-working-distance objective (magnification of 5, working distance of 14 mm). To avoid chromatic differences, the sample was illuminated by a monochromatic LED. Light passed through a lens into the zoom objective (zoom range from 0.9× to 10×), and the sample image was projected onto a CCD color camera (GigE camera, resolution 1280 × 1024). The camera and display provide additional electronic magnification: the total magnification of the sample image is about 300 to 3300 times. The display system reduces the operator-related deviations that occur during direct observation through the eyepiece [20]. The PEEK sample is precisely positioned between the DAC’s two diamond anvils. The optical microscope focal length is adjusted so that the PEEK sample is clearly imaged in the middle of the display screen to capture the appearance of PEEK under this pressure.

### 2.2. DAC and Sample Preparation

In our experiments, a high pressure was generated using a symmetric DAC featuring diamond anvils with a culet diameter of ~300 μm. For each DAC, the sample chamber was meticulously formed by pre-indenting a rhenium gasket and drilling a hole of 120 μm in diameter in the center of the indentation. A sample, roughly 70 μm in size, was placed into the DAC for pre-pressing. A tiny ruby ball, approximately 10 μm in diameter, was placed next to the sample, serving as a pressure gauge to determine the pressure from the ruby fluorescence [21] in units of GPa. Silicon oil, with relatively large molecules, can prevent the pressure-transmitting medium (PTM) from entering the free space of PEEK and affecting the EOS measurement of PEEK [22]. Therefore, silicone oil was used as the PTM, ensuring a uniform pressure distribution across the sample and facilitating accurate pressure measurements within the DAC. 

A thin PEEK film was smoothly attached to the adhesive tape, and then femtosecond laser processing was used to cut out small circular pieces with microscale diameters. The PEEK film was thin enough to easily cut through with a laser. At the same time, it was necessary to adjust the process parameters of laser cutting to ensure that the tape was not cut too much. The diameter of the PEEK microcircular pieces can be controlled by controlling the trajectory of laser scanning through the laser processing equipment. After a microcircular piece was cut, it was separated from the initial PEEK film to be processed and was independently adhered to the tape. After waiting for the laser to cut all the microdiscs, the initial PEEK film to be processed adhered to the tape was torn off from the tape, leaving individual PEEK microcircular pieces attached to the tape. After obtaining PEEK microcircular pieces, they were placed together with adhesive tape in a plasma-sputtering apparatus for gold plating.

### 2.3. Sample Area Measurements

For each pressure point, after setting the microscope to a 3300× magnification, the focus was fine-tuned so that the edges of the sample were clearly visible. Once the sample was clearly visible, images were captured and recorded. The images were then processed with the software Image J V1.54p [23], which automatically recognized and outlined the edges of the sample area. The area with an initial pressure of 0 GPa was selected as the atmospheric-pressure area, *S*_0_, of the PEEK. To ensure the reliability of the measurement results, at least three independent measurements were taken on the same sample, and the average value was taken as the final result. 

### 2.4. EOS Determination

(1) $$\left(\frac{V}{V_{0}}\right)={\left(\frac{S}{S_{0}}\right)}^{3/2}$$

where *V* is the sample volume at a given pressure, *V*_0_ is the sample volume at the ambient pressure, *S* is the average area at a given pressure, and *S*_0_ is the average area at the ambient pressure. To determine the bulk modulus (*K*_0_) and its derivative ($K_{0}^{\prime }$), the pressure–strain data, obtained by optical measurements, were fitted to the third-order Vinet EOS [24]:


(2) $$\begin{array}{c} P=3K_{0}\cdot {\left(\frac{V}{V_{0}}\right)}^{-2/3}\cdot \left[1-{\left(\frac{V}{V_{0}}\right)}^{1/3}\right]\cdot \exp\left[\frac{3}{2}\left(K_{0}^{\prime }-1\right)\cdot \left(1-{\left(\frac{V}{V_{0}}\right)}^{1/3}\right)\right]\\ =3K_{0}\cdot {\left(\frac{S}{S_{0}}\right)}^{-1}\cdot \left[1-{\left(\frac{S}{S_{0}}\right)}^{1/2}\right]\cdot \exp\left[\frac{3}{2}\left(K_{0}^{\prime }-1\right)\cdot \left(1-{\left(\frac{S}{S_{0}}\right)}^{1/2}\right)\right] \end{array}$$

## 3. Results and Discussions

**Figure 2:**
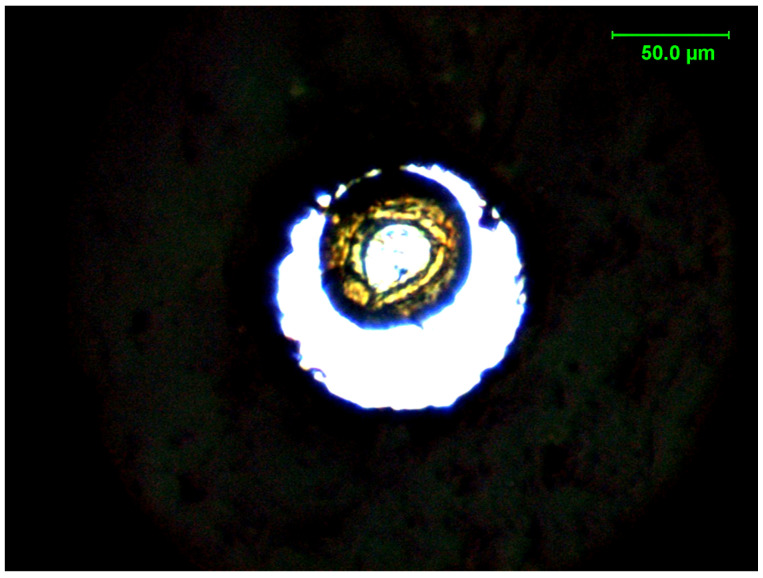
PEEK in the DAC was compressed up to 8 GPa.

In the case of the DAC experiments, PEEK was compressed up to 8 GPa at ambient temperature, and then optical images were collected. The area of PEEK was measured using the method mentioned in Section 2.3. The optical image of PEEK during compression up to 8 GPa is shown in Figure 2, which shows that the area of PEEK decreased with a volume contraction of about 20% upon compression from 0 to 8 GPa. 

**Figure 3:**
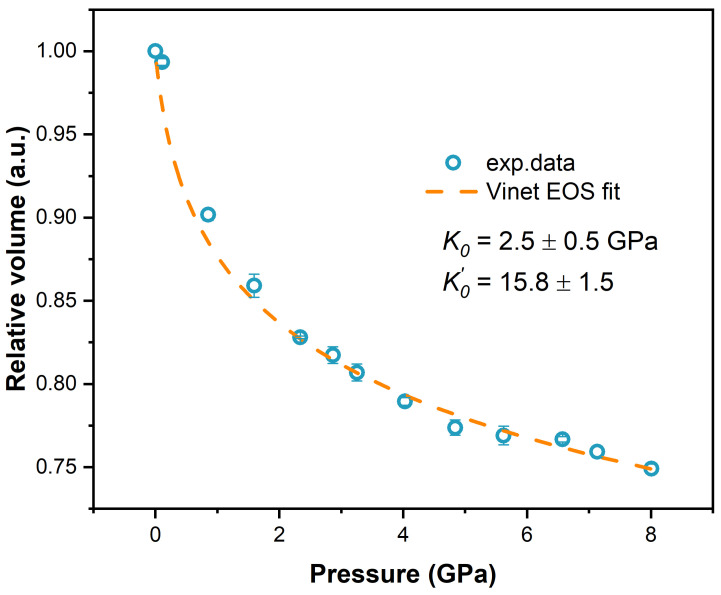
The relative volume of PEEK as a function of pressure during compression. The dashed line is the Vinet EOS fitting of PEEK.

The relative volume of PEEK as a function of pressure is shown in Figure 3. No obvious kinks were discovered in the experimental data points up to 8 GPa. The dominant error source in the data arises from the inappropriate focus of the sample, which will strongly affect the sample areas measured. In the present study, the focus of the sample can be tuned at the submicron level. In addition, we took three pictures at each pressure to minimize the focus errors. These *P*-*V* data can be fitted by the Vinet EOS, yielding *K*_0_ = 2.5 ± 0.5 GPa and $K_{0}^{\prime }$ = 15.8 ± 1.5. Previous experiments discovered that the measured Hugoniot data (*D*-*u_p_* plot) of PEEK are nonlinear at very low pressures, indicating a compressive behavior change [25,26]. The underlying mechanism is that the “free volume” of the amorphous part is compressed first until the density of the amorphous part reaches its crystalline counterpart. At higher pressures, there is a slope change in the Hugoniot data of PEEK, the reason for which is still under debate: phase transition or decomposition. However, these phenomena are not found in the present quasistatic compression experiment, demonstrating that the strain rate and/or temperature may affect the compressive behavior of PEEK at low pressures, which needs further investigation.

The EOS parameters determined for PEEK in this study, specifically the bulk modulus (*K*_0_) and its pressure derivative ($K_{0}^{\prime }$), provide a comprehensive understanding of its thermodynamic behavior under high pressure. To contextualize these findings, it is necessary to compare them with the EOS parameters of other polymers reported in the literature, as listed in Table 1. For instance, the EOS of polydimethylsiloxane (PDMS), as reported by Dattelbaum, D.M. et al. [27], shows a bulk modulus (*K*_0_) of 3.82 GPa, which is significantly larger than the 2.5 GPa observed for PEEK in this study. This difference in compressibility may be attributed to the distinct chemical structures and intermolecular interactions present in PEEK and PDMS. Additionally, the EOS parameters of PEEK can be compared with those of other high-performance polymers such as poly-(4-methyl-1-pentene) (PMP) and Kel F-800 copolymer, which were studied by Haill, T.A. et al. [28] and Benjamin, A.S. et al. [15], respectively. These studies reported EOS parameters that also vary significantly, reflecting the diverse mechanical properties and applications of these polymers. The EOS of PMP, for example, exhibits a bulk modulus that changes drastically with pressure, indicating a complex response to compression, similar to what was observed in PEEK but with different magnitudes and pressure dependencies. The comparison of EOS parameters not only highlights the unique thermomechanical properties of PEEK but also underscores the importance of material selection in high-pressure applications. The higher bulk modulus and pressure derivative of PEEK, as compared to other polymers, suggest its superior resistance to compression and potential for use in high-performance environments where maintaining structural integrity under pressure is critical. 

The optical measurement method is an effective technique for determining the EOS of PEEK, but it has certain limitations. Firstly, this technology is primarily suited for opaque materials, potentially rendering it unsuitable for transparent materials. Secondly, this technique is valid at hydrostatic (quasi-hydrostatic at least) conditions; thus, it cannot be applied to very high pressures. Lastly, the optical imaging method does not have the capability to provide information about the microstructure of polymers, which requires XRD, neutron diffraction, and small-angel scattering techniques [29,30,31,32].

## 4. Conclusions

In this study, we conducted an in-depth analysis of the isothermal EOS of PEEK combining the DAC and optical imaging techniques. By conducting experiments in a pressure range of up to 8 GPa, we successfully collected optical images of PEEK during compression and measured its area changes. Through the accurate fitting of experimental *P*-*V* data by Vinet EOS, we obtained PEEK’s bulk modulus, which is essential for understanding PEEK’s thermodynamic behavior under high-pressure conditions.

Our study not only expands PEEK’s accessible thermodynamic space but also provides a reliable way to predict its performance under extreme conditions. In addition, we also comprehensively analyzed error sources that may affect the experimental results and took corresponding measures to reduce the impact of these errors. Therefore, our results are highly reliable and accurate, providing important theoretical support for material design and the performance optimization of PEEK in high-performance applications. Additionally, this method offers a rapid means of acquiring preliminary EOS information for polymers and other materials. It can be used to quickly provide approximate data for immediate incorporation into computational models and for planning dynamic plate-impact experiments. The study of polymer behavior under high pressures, along with the provision of new experimental data and analytical methods for EOS research, holds significant importance for understanding the behavior of polymers under extreme conditions and offers new directions for future research. Further research on PEEK’s isothermal EOS is expected to promote its wider application in high-end industrial and technological fields.

## Figures and Tables

**Table 1:** The Vinet EOS parameters of different polymers.

Polymers	Vinet EOS Parameters		Ref.
	*K*_0_	$K_{0}^{\prime }$	
PEEK	2.5	15.8	This work
PDMS	3.82	-	[27]
Kel F-800	14.6	8.0	[15]

## Data Availability

The original contributions presented in this study are included in the article. Further inquiries can be directed to the corresponding author.
